# Spectral-Spatial Differentiation of Brain Activity During Mental Imagery of Improvisational Music Performance Using MEG

**DOI:** 10.3389/fnhum.2018.00156

**Published:** 2018-04-24

**Authors:** Jared Boasen, Yuya Takeshita, Shinya Kuriki, Koichi Yokosawa

**Affiliations:** ^1^Graduate School of Health Sciences, Hokkaido University, Hokkaido, Japan; ^2^Faculty of Health Sciences, Hokkaido University, Hokkaido, Japan

**Keywords:** magnetoencephalography, improvisation, rhythm, music therapy, music performance, cognition

## Abstract

Group musical improvisation is thought to be akin to conversation, and therapeutically has been shown to be effective at improving communicativeness, sociability, creative expression, and overall psychological health. To understand these therapeutic effects, clarifying the nature of brain activity during improvisational cognition is important. Some insight regarding brain activity during improvisational music cognition has been gained via functional magnetic resonance imaging (fMRI) and electroencephalography (EEG). However, we have found no reports based on magnetoencephalography (MEG). With the present study, we aimed to demonstrate the feasibility of improvisational music performance experimentation in MEG. We designed a novel MEG-compatible keyboard, and used it with experienced musicians (*N* = 13) in a music performance paradigm to spectral-spatially differentiate spontaneous brain activity during mental imagery of improvisational music performance. Analyses of source activity revealed that mental imagery of improvisational music performance induced greater theta (5–7 Hz) activity in left temporal areas associated with rhythm production and communication, greater alpha (8–12 Hz) activity in left premotor and parietal areas associated with sensorimotor integration, and less beta (15–29 Hz) activity in right frontal areas associated with inhibition control. These findings support the notion that musical improvisation is conversational, and suggest that creation of novel auditory content is facilitated by a more internally-directed, disinhibited cognitive state.

## Introduction

To paraphrase Limb and Braun (2008), improvisational music performance is the spontaneous, on-line ideation and expression of novel melodic, harmonic and rhythmic musical elements within a relevant musical context. In a group context, improvisational music performance is furthermore thought to be synonymous with conversation (Monson, 1996). These ideational, expressive and conversational aspects of musical improvisation have made it an important tool in music therapy where it serves as a vehicle for communication between patient and therapist, both of whom use instruments to express themselves in an ongoing musical conversation. Moreover, the spontaneity of musical improvisation makes it intrinsically disinhibitory, which facilitates externalization of internal states. Thus, music therapy employing improvisation is thought to be particularly useful for connecting with patients who struggle with verbal, social, or emotional processing. Indeed, in patients with depression, autism spectrum disorder, or other forms of cognitive dysfunction, improvisational music therapies have been reported to improve well-being, along with communication, sociability, and emotional and creative expression (Kim et al., 2008; Erkkilä et al., 2011; Rylatt, 2012).

To understand these therapeutic effects, it is important to clarify the nature of brain activity associated with improvisational music performance. Results from functional magnetic resonance imaging studies (fMRI) using improvisational music performance tasks have provided important insight, and suggest hemispheric laterality of certain aspects of brain activity or function associated with improvisational music performance. Particularly notable is the study by Donnay et al. (2014) which used a communicative style of musical improvisation akin to that employed in improvisational music therapy, and observed bilateral activation of temporal brain areas associated with communication and left hemisphere activation of inferior parietal areas associated with sensorimotor integration. Donnay et al. (2014) also observed deactivation of frontal areas associated with executive processing. Frontal deactivation, concentrated in the right hemisphere, was also observed by Limb and Braun (2008), who proposed that it may be key to the spontaneity or disinhibition intrinsic to musical improvisation. Right hemispheric deactivation indicative of disinhibition was also found by Berkowitz and Ansari (2008) in the temporal parietal junction in musicians compared to non-musicians during improvisational music performance. Meanwhile, de Manzano and Ullén (2012) observed that functional activity between the left pre-motor area and the cerebellum increased during right-handed rhythmic compared to melodic improvisational music performance.

Electroencephalographic (EEG) studies have also made valuable contributions. Some have identified frequency bands and oscillatory characteristics of interest during musical improvisation. For example, Lopata et al. (2017) proposed increased oscillatory alpha power during musical improvisation compared to non-improvisational performance as a sign of increased spontaneous or bottom-up processing. Dikaya and Skirtach (2015) found higher levels of theta oscillatory coherence between left temporal-frontal electrodes, and higher beta spectral power over left frontal sensors during musical improvisation compared to non-improvisational musical performance. Meanwhile, Müller et al. (2013) found greater inter-brain synchronization of theta and delta oscillatory activity compared to higher frequency oscillatory activity during musical improvisation between two guitarists. Additionally, Adhikari et al. (2016) attributed decreased coherence with frontal oscillatory brain activity during musical improvisation compared to during performance of pre-learned music as important to the spontaneity of improvisation. Network-based analysis has also been used to identify potential regions of interest during musical improvisation. For instance, Wan et al. (2014) used causality analyses, and found that the frontal, parietal and temporal regions were important for differentiating between brain activity during improvisational and non-improvisational music performance.

These neurophysiological studies shed light on the nature of brain hemodynamics and spontaneous oscillatory activity during improvisational music performance. However, how brain activity in different oscillatory frequency bands is modulated in different brain areas due to improvisational music performance remains unclarified. To this end, studies employing magnetoencephalography (MEG), which permits spectral-spatial analyses of brain activity, would be well suited. Some MEG studies regarding music performance have been reported, such as one regarding mu rhythm suppression due to finger tapping on a drum (Caetano et al., 2007), and another regarding rhythmic brain activities related to singing (Gunji et al., 2007). However, we could find no reports regarding improvisational music performance in MEG.

Improvisational music performance inherently involves physical movement. However, it is known that physical movement affects MEG (and EEG) recording (Gross et al., 2013). To avoid this confound when the brain activity pertaining to physical action is desired, neurophysiological studies will often record brain activity during mental imagery of the physical action of interest. Although the degree to which brain activity during mental imagery corresponds with that during real action is an area of continuing research (Pearson et al., 2015), the existence of correspondence is undeniable. As far as mental imagery of music and actual listening are concerned, numerous overlapping areas of brain activation have been shown to be involved including: bilateral auditory antereolateral belt, Wernicke’s area, and intraparietal sulcus; and left premotor cortex and supplementary motor area (Zhang et al., 2017). During physical music performance, mental imagery of the played audio and actual listening of the played audio has been shown it to exhibit similarities with respect to modulation and cortical location of high frequency brain activity (Martin et al., 2017). Mental imagery of music performance has also been shown to reflect the structure of the imagined music by modulating in accordance with targeted beat and meter frequency (Okawa et al., 2017). Mental imagery of improvisational music performance was found to exhibit brain activity in the occipital lobe that correlated highly with that exhibited during passive listening of the subject’s own prior improvisational music performance (Sanyal et al., 2016). Additionally, brain activity during mental imagery of music performance and that during actual performance has been shown to share numerous causal network connections (Adhikari et al., 2016). Collectively, these findings indicate that brain activity during mental imagery of music perception and music performance (improvisational or otherwise) is relevant to and shares many commonalities with brain activity during actual perception and performance. Thus mental imagery is an insightful and useful design strategy for neurophysiological experimentation.

With the present study, we therefore used an experimental paradigm incorporating mental imagery to demonstrate the feasibility of improvisational music performance experimentation in MEG. First, we designed an MEG-compatible keyboard. Then, we used this keyboard with musicians experienced at improvisation to explore and differentiate brain areas and spontaneous brain oscillatory modulation associated with mental imagery of improvisational music performance. As for musical elements that can be improvised, pitch, tone, rhythm, and loudness are all conceivable options. However, rhythm is arguably the most fundamental musical element, and hence it is used extensively in improvisational music therapy (Montello and Coons, 1998; Burns et al., 2001; Rickson and Watkins, 2003). Provided that the metric structure of the music is fixed (i.e., a set tempo is used), rhythmic improvisation equates to free execution of the number of notes in congruence with the metric structure, within a given time-frame (e.g., one measure). In this way, our experimental paradigm focused on differences due to improvisation or non-improvisation of just the musical element of rhythm. In consideration of oscillatory frequencies shown by EEG studies to be relevant during improvisational cognition, our analyses of brain activity focused on the theta (5–7 Hz), alpha (8–12 Hz), and beta (15–29 Hz) bands. Additionally, considering the hemispheric laterality of certain results from prior fMRI studies, we furthermore focused our analyses on the left and right hemispheres separately.

## Materials and Methods

### Subjects

This study targeted active musicians with improvisational experience in the Sapporo metropolitan area of Hokkaido prefecture, Japan. Subjects were recruited via flyer postings at our institution and via online social media. Subjects’ playing frequency and frequency of improvisation were assessed via a music experience questionnaire modeled after that used by Bashwiner et al. (2016). Thirteen right-handed subjects (10 males; mean ± SD age, 35.7 ± 8.6 years) with improvisational playing experience were selected. Eleven subjects practiced improvisation on a weekly to daily basis. Two subjects practiced improvisation on a monthly basis. All subjects could improvise readily when prompted and could easily perform the tasks in this study. Subjects’ practice frequency with their primary instrument ranged from several times weekly to several hours or more daily. Only one subject played the piano as her primary instrument. For further details regarding the characteristics and musical experience of subjects, please see Supplementary Table S1. Written informed consent was obtained from all subjects prior to participation in this study, which was approved by the Ethics Committee of the Faculty of Health Sciences and the Ethics Committee of the Graduate School of Medicine, Hokkaido University, and conformed to the 1964 Helsinki declaration and its later amendments or comparable ethical standards.

### Keyboard and Auditory Feedback

We constructed an MEG-compatible keyboard with five keys whose depression activated individually-placed, circular Piezo sensors. Serial signals from the Piezo sensors were fed outside the shield room into an Arduino circuit board connected to a notebook PC. An open-source program was used to convert individual Piezo sensor signals into MIDI. This program was purposely modified to eliminate velocity effects of Piezo sensor activation. In other words, regardless of the strength a key was depressed, the loudness of the sound generated from its activation was uniform across keys. MIDI signals from each key were further programmed to play a major pentatonic scale beginning from the leftmost key with middle C (C3; 261.6 Hz). Free software was then used to feed these MIDI signals into a virtual MIDI port (Hairless) that was then read by music production software and played through a native MIDI piano instrument plugin (Ableton Live 8). Piano sound output was routed to an electrostatic speaker within the shield room to provide subjects with auditory feedback of their performance. The latency between Piezo sensor activation and audio output was manually set to 16 ms, a time which simultaneously did not burden the processing speed of the PC used, and permitted natural musical performance by the subject.

### Experimental Design and Procedure

The present study was designed with two types of tasks, Single-finger and Multi-finger. Each task type comprised two response conditions, Copy and Improvise. In Single-finger Copy, the subject monotonically copied the rhythm of the stimulus using a single finger. In Single-finger Improvise, the subject monotonically improvised a novel rhythm in response to the stimulus using a single finger. In Multi-finger Copy, the subject polytonically copied the rhythm of the stimulus using any combination of fingers. In Multi-finger Improvise, the subject polytonically improvised the rhythm via any combination of fingers. The underlying difference between Copy and Improvise in each task was improvisation or non-improvisation of rhythm.

The experiment was performed in two sessions. Each session consisted of one block of eight stimulus-response epochs for each task condition, with block order randomized between subjects. Within any given block, stimulus-response epochs were presented with no interruption to the musical continuum (i.e., they were presented continuously with no interval or jitter between epochs). Each stimulus-response epoch corresponded to a unique polytonic keyboard stimulus pattern composed of the same five notes, with identical tonal properties, as those produced by the MEG keyboard. There were thus a total of 16 stimulus patterns. The exact notes used and their number varied with each pattern. The same stimulus patterns were used for each task/condition, and presented in the same order for each block in each session.

**Figure 1:**
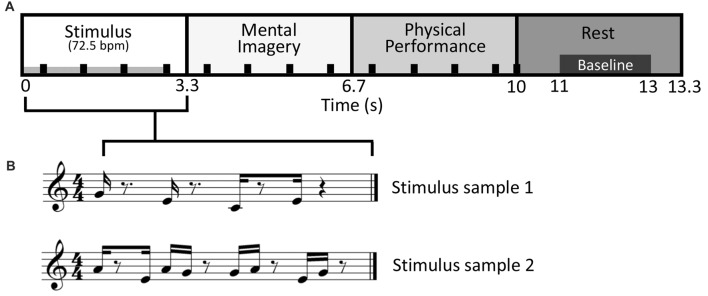
Stimulus-response design. **(A)** Diagram of the stimulus-response epoch, a variation on “trading fours”. Sixteen different stimulus patterns were used. The duration of stimulus presentation is indicated by the gray bar in the lower left of the stimulus period. Responses during the Mental Imagery and Physical Performance periods were respectively done via mental imagery and on the magnetoencephalography (MEG) compatible keyboard. The black tick marks denote the backbeat that was present during the first three periods, ending on the first beat of the rest period. **(B)** Two sample stimuli patterns shown in the time frame of the Stimulus period, in musical notation for representative purposes only.

The stimulus-response epoch was designed to comprise four musical measures in 4/4 time at a tempo of 72.5 bpm with a total length of 13.3 s. In the first measure (0–3.3 s; hereafter, stimulus period), one of the polytonic keyboard stimulus patterns was presented via an electrostatic speaker in the shielded room. In the second measure (3.3–6.7 s; hereafter, mental imagery period), subjects performed their response to the stimuli according to the given condition via mental imagery. In the third measure (6.7–10.0 s; hereafter, physical performance period), the notes that were mentally imagined were recalled and physically performed on the MEG keyboard. In the fourth measure (10–13.3 s; hereafter rest period), the subjects rested. A 2 s portion of the rest period (11–13 s) was used for calculating baseline activity. Meanwhile, a percussive quarter note backbeat played throughout the first three periods, ending on the first beat of the fourth period. The backbeat served to help subjects maintain the timing-accuracy of their responses (see Figure 1).

This design permitted musical performance akin to a commonly used improvisational structure in jazz and blues called “trading fours,” in which two musicians take turns improvising and conversing to one another via their instruments. A similar design was used by Donnay et al. (2014). Conventionally, a “trading fours” structure would only comprise a stimulus period (i.e., a musical phrase played by another musician), and a physical performance period (i.e., a musical phrase played by oneself in response to the musical phrase played by the other musician). The design used in the present study maintained the conversational structure of “trading fours,” albeit with modifications to facilitate MEG experimentation. Specifically, the mental imagery period permitted analysis of brain activity that was free from noise or confounds due to physical movement. The mental imagery period was also the only time where cognition related to improvisational performance was occurring. Thus, the present study focused exclusively on comparisons of brain activity between conditions during the mental imagery period. The physical performance period served merely as a means for measuring behavior response (see “Behavioral Data Collection” section), and keeping the subjects enjoyably and musically engaged. The rest period meanwhile provided a reference frame to which the modulation of brain activity could be normalized (i.e., it permitted calculation of baseline activity levels).

Prior to the experiment, subjects were given detailed instructions on how to perform the experimental tasks and conditions during a training session conducted outside the shielded room. During the training session, a series of continuous stimulus-response epochs were played over two external computer speakers while subjects sat upright in a chair. During the physical performance period of each stimulus-response epoch, subjects used their right hands to tap an iPad running a digital keyboard application (Garage Band) which was programmed to a C3 major pentatonic scale (i.e., the iPad mimicked the MEG keyboard that subjects would use during the actual experiment inside the shielded room). Subjects were instructed to fix the performance of each finger to just one key on the scale (i.e., the thumb played C3, the index finger played D3, etc.). They were also specifically instructed to not move their heads, trunk, or other extremities, and to move their right hands for performance during the physical performance period only. Once it was clear that subjects understood the instructions and could perform the experiment without difficulty, they were prepared for MEG recording.

### MEG Recording and Processing

All MEG measurements were done within a magnetically shielded room using a 76 ch. custom-type helmet MEG system (Elekta-Neuromag). Head position indicators, fiducials, and head points were digitized according to standard MEG operating procedure (Hansen and Kringelbach, 2010). The subject was positioned in an upright position in the MEG measurement chair, onto which a table was attached. Upon the table, the MEG keyboard was fixed with tape at a comfortable position for right-handed performance. The subjects then placed their right hand in position on the keyboard, with their palm resting on the table, and each fingertip resting on the surface of its corresponding key. A box was then placed over top of the keyboard and their right hand to prevent visual distraction from hand and finger movement. Subjects were further instructed to look straight ahead at a marker fixed on the shield room wall. They were also re-instructed to move only their right hand, and only during the physical performance period. At all other times, they were instructed to rest the fingers of their right hand on the surface of the keys, and their right palm on the surface of the table. Throughout the experiment, the subject was visually and aurally monitored to ensure comfort and compliance with all experimental instructions. Prior to the start of each experimental block, the experimenter used an external microphone connected to the electrostatic speaker in the shielded room to communicate the relevant forthcoming stimulus-response condition, and to verify that the subject understood the appropriate response for that condition.

MEG signals were band-pass filtered from 0.6 Hz to 200 Hz and recorded at a 600 Hz sampling frequency. All MEG data processing was performed in Brainstorm^1^. This processing began with removal of noisy or dead channels. Components of physiological artifacts and periodic noise were isolated and removed using independent component analysis. A comb filter was applied at 50 Hz and related harmonic frequencies to remove line noise. A band-pass filter was then applied from 1 Hz to 40 Hz. Cleaned and filtered data was then epoched at −1 s to 14.3 s relative to stimulus onset. Each epoch was visually scanned, and those with movement artifacts were removed. Subject head points and fiducials were coregistered to a common template brain. An overlapping-sphere forward model was computed, and minimum-norm estimation was used to calculate cortical currents without dipole orientation constraints. To facilitate exploratory analyses, the cortical surface was divided into hemispheres and each hemisphere parcellated into 34 areas based on the Desikan-Kilany cortical surface atlas. The time-series of cortical currents in each brain area was decomposed into the theta (5–7 Hz), alpha (8–12 Hz) and beta (15–29 Hz) frequency bands, and their corresponding envelopes computed using Hilbert transform. Time-frequency envelopes in each frequency band in each brain area were averaged across epochs within subjects for each condition. The amplitude of the time-frequency envelopes was standardized across subjects as a percent deviation from baseline using the following equation where x is the amplitude of the time-frequency envelope at each time point, and μ is the time-average over the baseline period (11–13 s, see Figure 1).

$$X_{\text{std}}=\frac{x-\mu }{\mu }\times 100$$

Standardized time-frequency envelopes were averaged over the mental imagery period in each brain area for each frequency band for each subject. Resulting values were used in statistical analyses.

### Behavioral Data Collection

Although the present study expressly focused on brain activity during the mental imagery period, a time when there was no behavioral response, the experiment was designed such that the notes imagined during the mental imagery period are recalled and physically played during the physical performance period. Thus, we assumed that the notes played during the physical performance period were a reasonable representation of the behavioral response during the mental imagery period. As the number of notes imagined and correspondingly physically played (hereafter, note count) in each epoch was not controlled in this study, it is conceivable that note count may have affected brain activity during the mental imagery period. To assess this, concurrent with MEG recording, keyboard responses during the physical performance period were recorded for each subject in the form of MIDI data. From this MIDI data, mean note counts in each task and condition for each subject were calculated for use in statistical analyses.

### Statistical Analyses

Mean note counts for Copy and Improvise were contrasted with the mean stimulus note count across all 16 stimulus patterns via one-sample *t* tests to assess behavioral adherence to the task conditions. Additionally, differences in mean note count between Copy and Improvise were analyzed using paired *t* tests. Mean standardized brain activity over the mental imagery period in each hemisphere in each frequency band of interest was analyzed using two-way repeated measures analysis of variance (RM ANOVA) ((brain area: 34 areas per hemisphere) × (condition: Copy, Improvise)). Homogeneity of data from each hemisphere was assumed based on Levene’s tests. In cases where RM ANOVA revealed brain areas with significant differences between conditions, the relationship of performance note count to the level of frequency-specific brain activity in that corresponding area was analyzed using Pearson’s correlation analyses (within and across conditions) and multiple regression analyses (with note count and condition as regressors). All statistical tests were two-tailed and conducted using SPSS (IBM), with significance determined at *p* ≤ 0.05.

## Results

**Figure 2:**
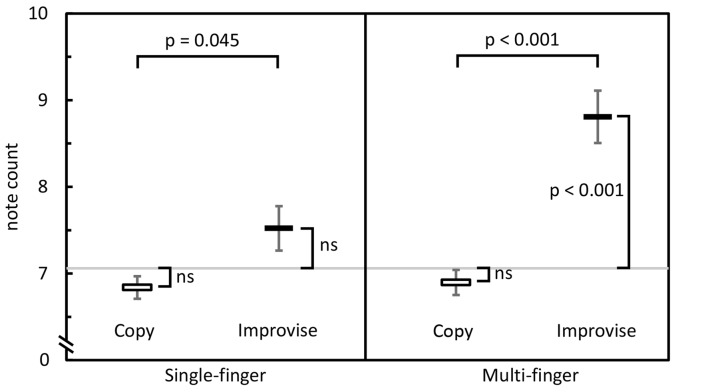
Behavioral comparisons. Mean note counts for Copy and Improvise in the Single-finger and Multi-finger tasks (*N* = 13). Results indicated increased rhythmical freedom in subject responses during Improvise conditions, and a high degree of accuracy during Copy conditions. The horizontal gray line represents the mean note count across all 16 stimulus patterns (7.06).

In the Single-finger task, one-sample *t*-tests revealed that note counts for Improvise (mean ± SE, 7.587 ± 0.257) and Copy (mean ± SE, 6.803 ± 0.130) were not significantly different from the mean note count for all 16 stimulus patterns (7.06 notes; *p* = 0.072 and *p* = 0.063, respectively), indicating that responses in neither condition deviated significantly from the level of rhythmic complexity in the stimulus patterns. This result also implied a high degree of accuracy for Copy responses. Meanwhile, the fact that mean note count and standard error were larger for Improvise indicated a tendency towards expression of increased rhythmic freedom, a notion that was corroborated by the paired *t*-test which revealed that note counts for Improvise were significantly greater compared to Copy (*p* = 0.045; see Figure 2 left panel). As for brain activity in the Single-finger task, RM ANOVA revealed no significant effects of condition nor interactions between brain area and condition in any frequency. As such, our report will hereafter focus on results from the Multi-finger task.

**Figure 3:**
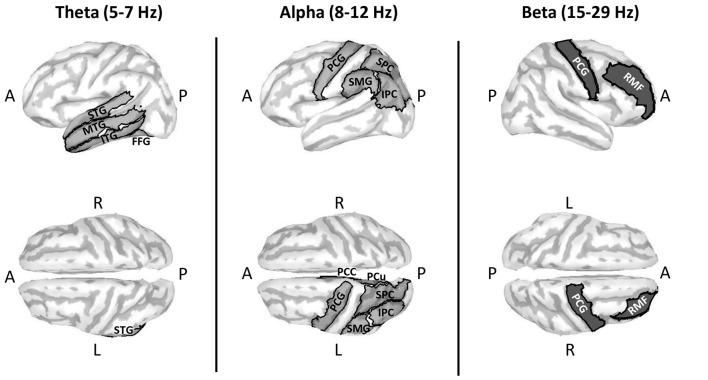
Cortical maps highlighting areas with significantly different oscillatory activity between conditions for the Multi-finger task. Compared to Copy, Improvise induced mean current density strengths over the mental imagery period that were higher in the theta band in the left temporal cortex, higher in the alpha band primarily in the left posterior parietal cortex and lower in the beta band in right prefrontal areas. Improvisational cognition was thus differentiated according to the frequency of oscillatory activity in non-overlapping brain regions. A, P, L and R, respectively denote anterior, posterior, left and right. For further details, see Table 1.

**Figure 4:**
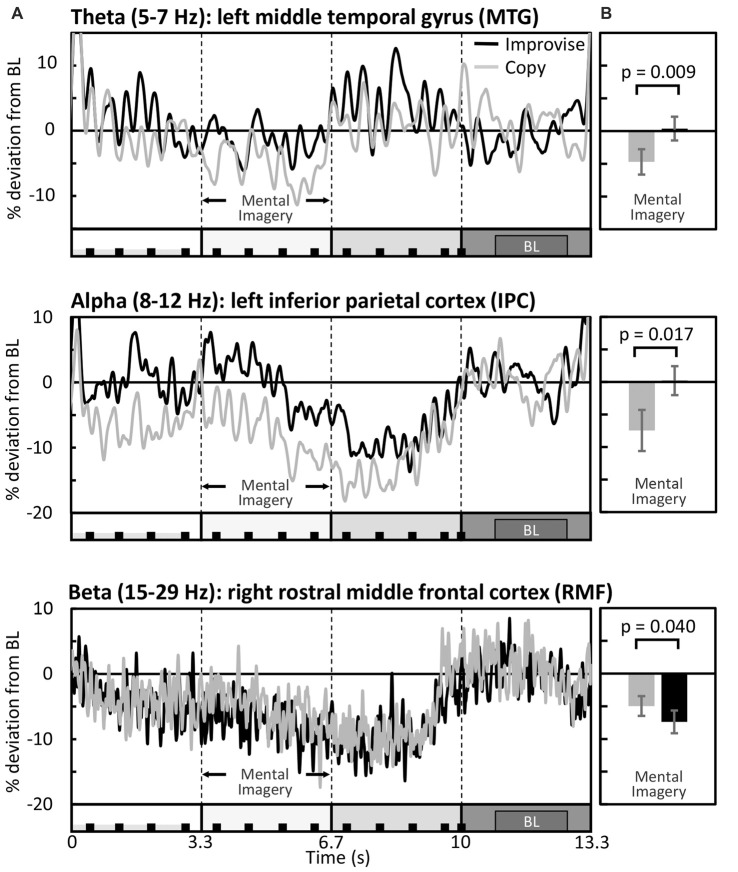
Differential modulation of spectral activity during the Multi-finger task. **(A)** Mean brain activity across the entire stimulus-response epoch in representative brain areas (*N* = 13). The X axis is represented by a minimalized version of the stimulus-response epoch diagram which is detailed in Figure 1. BL denotes the baseline period. **(B)** Respective mean activity over the mental imagery period.

RM ANOVA of theta band activity during mental imagery in the left hemisphere revealed no significant main effect of area or condition (*F*_(33,396)_ = 1.280, *p* = 0.143; *F*_(1,12)_ = 2.103, *p* = 0.173; respectively). However, there was a significant interaction between area and condition (*F*_(33,396)_ = 1.763, *p* = 0.007). Simple main effects tests revealed greater levels of activity for Improvise than Copy in the left: fusiform gyrus (FFG; *p* = 0.047), inferior temporal gyrus (ITG; *p* = 0.023), middle temporal gyrus (MTG; *p* = 0.009), superior temporal gyrus (STG; *p* = 0.030), and parahippocampal gyrus (PHG; *p* = 0.049). In the right hemisphere, there was a significant main effect of area (*F*_(33,396)_ = 1.725, *p* = 0.009), but no main effect of condition nor interaction between area and condition (*F*_(1,12)_ = 0.581, *p* = 0.461; *F*_(33,396)_ = 0.93, *p* = 0.59; respectively). Thus, areas having significantly different theta activity were concentrated in the left temporal cortex (see Figure 3 left). A plot of representative mean theta activity across subjects in the left MTG reveals that theta band activity for Improvise was strongly modulated in correspondence with the stimulus and physical performance periods, and that it remained higher compared to Copy throughout the mental imagery period (Figure 4 top panel).

In the alpha band, results in the left hemisphere revealed a significant main effect of area (*F*_(33,396)_ = 3.653, *p* < 0.001), a marginal main effect of condition (*F*_(1,12)_ = 4.553, *p* = 0.054), and a significant interaction between area and condition (*F*_(33,396)_ = 2.080, *p* = 0.001). Simple main effects tests revealed significantly greater levels of activity for Improvise than Copy in the left: precentral gyrus (PCG; *p* = 0.019), superior parietal cortex (SPC; *p* = 0.017), inferior parietal cortex (IPC; *p* = 0.017), supramarginal gyrus (SMG; *p* = 0.045), precuneus (PCu; *p* = 0.021) and posterior cingulate cortex (PCC; *p* = 0.040). In the right hemisphere, results revealed no main effects of area or condition, nor interaction between area and condition (*F*_(33,396)_ = 1.297, *p* = 0.131; *F*_(1,12)_ = 0.508, *p* = 0.49; *F*_(33,396)_ = 0.698, *p* = 0.90; respectively). Thus, areas having significant different alpha band activity did not overlap with those relevant to the theta band, were also left hemispheric, and were predominantly concentrated in the posterior parietal cortex, which comprises the SPC, IPC, SMG, and PCu (see Figure 3 middle). A plot of representative average alpha activity in the left IPL (Figure 4 middle panel) reveals that activity for both condition exhibited desynchronization dynamics during the mental imagery period similar to that which many studies have observed during idea generation and periods of planning prior to physical movement (Caetano et al., 2007; Deiber et al., 2012; Schwab et al., 2014; Fumuro et al., 2015). Activity for Improvise was higher however throughout the stimulus period and well into the mental imagery period, where it remained at or above baseline levels until dropping in preparation for physical performance.

In the beta band, results in the left hemisphere revealed a significant main effect for area (*F*_(33,396)_ = 3.886, *p* < 0.001), but the main effect of condition and interaction between area and condition were not significant (*F*_(1,12)_ = 0.227, *p* = 0.64; *F*_(33,396)_ = 1.284, *p* = 0.140; respectively). In the right hemisphere, there was a significant main effect of area (*F*_(33,396)_ = 3.886, *p* < 0.001), but no main effect of condition (*F*_(1,12)_ = 0.812, *p* = 0.39). However, there was a weakly significant interaction between area and condition (*F*_(33,396)_ = 1.473, *p* = 0.048). Simple main effects tests revealed significantly lower levels of activity for Improvise than Copy in the right: rostral middle frontal cortex (RMF; *p* = 0.040), and the PCG (*p* = 0.045; see Figure 3 right). A plot of representative average beta activity in the right RMF reveals that activity steadily decreased for both conditions from the stimulus through the performance periods. However, lower activity for Improvise is apparent, particularly during early mental imagery (Figure 4 bottom panel).

Table 1, summarizes the results from significant interactions and simple main effects as per the RM ANOVAs for the Multi-finger task.

**Table 1:** Multi-finger task repeated measures analysis of variance (RM ANOVA) results summary.

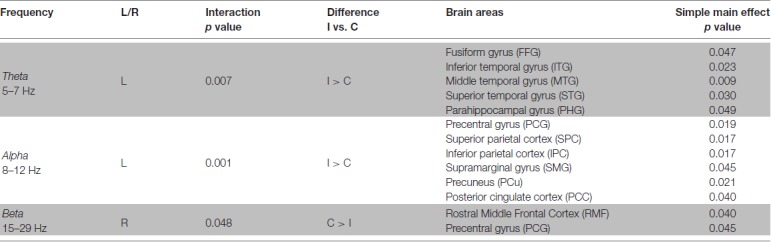

*L, R, I and C refers to left, right, Improvise and Copy, respectively*.

**Figure 5:**
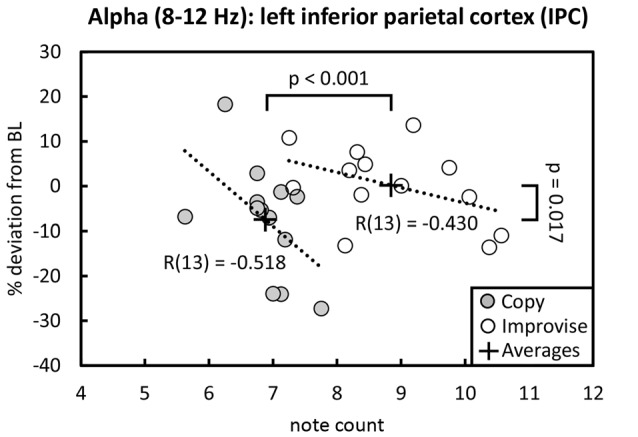
Alpha activity vs. note count. Improvise exhibited higher levels of alpha activity than Copy despite having higher note count, and despite a trend within each condition towards lower alpha levels with increasing note count. Trend lines are based on Pearson’s correlation analyses.

Behaviorally in the Multi-finger task, note counts for Improvise (mean ± SE, 8.841 ± 0.302) were significantly higher than Copy (mean ± SE, 6.880 ± 0.145; *p* < 0.001), and significantly greater than the mean note count for all 16 stimulus patterns (*p* < 0.001), again reflecting expression of increased rhythmical freedom during Improvise. Meanwhile for Copy, standard error was less than that for Improvise, and note count was not significantly different from the mean note count for all 16 stimulus patterns, implying a high degree of accuracy during Copy responses (see Figure 2 right panel). Pearson’s correlation analyses revealed no significant relationship between note count and brain activity in any frequency band, in any area, within or across conditions. Nevertheless, multiple regression analyses revealed that note count was predictive of brain activity in the alpha frequency band in the left IPC (*F*_(2,23)_ = 4.207, *p* = 0.028, *R*^2^ = 0.268) and the left PCC (*F*_(2,23)_ = 3.439, *p* = 0.049, *R*^2^ = 0.230). Standardized beta coefficients for the contribution of note count in these two areas were respectively *β* = −0.593 (*p* = 0.044) and *β* = −0.663 (*p* = 0.029), indicating a trend towards decreased alpha band brain activity with higher note count. However, the standardized beta coefficients for the contribution of condition in these two areas were respectively *β* = 0.806 (*p* = 0.008) and *β* = 0.730 (*p* = 0.017), indicating the greater importance of condition over note count at predicting alpha activity, and corroborating the RM ANOVA finding that alpha activity levels are higher for Improvise than for Copy. Figure 5 helps illustrate these multiple regression findings, using Pearson’s correlation results for alpha band activity vs. note count within conditions at the left IPC. The figure indicates that alpha activity levels are higher for Improvise than Copy despite trends towards lower alpha activity with higher note count within each condition.

## Discussion

The present exploratory study sought to differentiate brain areas and oscillatory frequency bands relevant to improvisational cognition by comparing brain activity during mental imagery of music performance in conditions where rhythm was either improvised or copied. Our analyses of the Multi-finger task revealed significant interactions between condition and brain area, where significant differences in brain activity were found between conditions for all three frequency bands of interest. Moreover, the brain areas where significant differences in brain activity were found differed depending on the oscillatory frequency band.

We found that theta band activity was significantly greater for Improvise compared to Copy comprehensively throughout the left temporal cortex in the STG, MTG, ITG, FFG and PHG. Numerous studies have shown that event-related increases in theta activity localized within the temporal cortex including within the STG are important for auditory rhythm processing (Ahissar et al., 2001; Ghitza, 2012; Luo and Poeppel, 2012; Hyafil et al., 2015). The left STG in particular has been implicated as important for the production of syllables and discreet rhythm elements of speech (i.e., sublexical speech; Hickok and Poeppel, 2004), implying a functional role in rhythm-based auditory production. Additionally, activation of temporal areas, including the STG, was observed by Donnay et al. (2014) during a similar style of musical improvisation, and has consistently been demonstrated during mental imagery of music (Halpern and Zatorre, 1999; Kraemer et al., 2005; Groussard et al., 2010; Zvyagintsev et al., 2013). During the Improvise condition of the present study, the need to improvise novel rhythm patterns likely placed more demand on rhythmic production processing, and may thus explain the greater theta activity found for Improvise at the STG.

Areas other than the STG where theta activity was significantly higher for Improvise may be functionally important to the improvisational nature of the rhythm production. For example, the ITG and MTG in the left hemisphere specifically, and the FFG, are all reportedly important for linguistic semantic processing (Chee et al., 1999; Hickok and Poeppel, 2000; Balsamo et al., 2006). Left anterior temporal areas, including the left MTG, have also been linked to musical semantic processing (Platel et al., 2003). Theta power increases over left temporal areas are associated with increased demand on linguistic semantic processing (Bastiaansen et al., 2005), and may likewise occur with increased demand on musical semantic processing. Meanwhile, the PHG has been implicated in emotional processing, including that related to music (Koelsch, 2005; Aminoff et al., 2013). Processing the semantic and emotional context of sounds is arguably an intrinsic part of auditory communication, and likely more important for Improvise than Copy due to the conversational style of improvisation used. Thus, the higher theta activity found for Improvise may comprehensively reflect an increased demand on rhythmic communication processing.

Memory processing is also essential for both Improvise and Copy during the mental imagery period of the present study. This is because mentally imagined musical information must be memorized for playback during the physical performance period. Note count could be considered an index of memory load during the mental imagery period. Increasing memory load reportedly results in lower alpha activity (Stipacek et al., 2003). In agreement with this, the contribution of note count found via multiple regression analyses indicated decreased alpha band activity with greater note count at the left IPC and PCC, areas known to be functionally important in memory tasks (Maddock et al., 2001; Koenigs et al., 2009). Based on the above, the fact that note count was significantly greater for Improvise than Copy suggests that memory load was higher for Improvise, and thus favors it having lower alpha band activity. Nevertheless, alpha band activity was significantly greater for Improvise than Copy. Moreover, contribution of condition via multiple regression analyses indicated the greater importance of condition over note count. This suggests that the difference in alpha activity between conditions was being influenced apart from memory by another more important aspect of cognition.

We propose this other aspect of cognition to be its directionality with respect to the imagined music performance in each condition. This notion is supported by the fact that the left SMG, IPC, SPC, PCu and PCG, areas where significant differences in alpha activity were found, are all contralateral to the hand used in imagined music performance. Additionally, these areas have reported links to auditory-related sensorimotor integration (Knight et al., 1989; Wolpert et al., 1998; Rushworth et al., 2001; Bangert et al., 2006; Koenigs et al., 2009), and mental imagination of motor activity (Kawashima et al., 1994; Porro et al., 1996; Caminiti et al., 1998; Simon et al., 2002; Lotze and Halsband, 2006; Sacco et al., 2006). During tasks employing mental imagination, divergent thinking, and analytical problem solving, alpha power is higher when cognition is internally-directed compared to when it is externally-directed (Cooper et al., 2003; Kounios et al., 2008; Benedek et al., 2014; Kounios and Beeman, 2014). Alpha power has also been found with EEG to be higher during musical improvisation compared to other musical tasks. This result was likewise attributed to the cognition involved being more bottom-up or internally-directed (Lopata et al., 2017). Indeed, internally-directed cognition is thought to be an intrinsic aspect of tasks such as musical improvisation which require generation of novel content (Beaty, 2015). In line this, we think cognition is directed internally during the mental imagery period of the Improvise condition to produce novel rhythm responses, rather than externally to ensure accurate duplication of rhythm patterns as in the Copy condition. Thus we speculate that the higher alpha activity for Improvise is a sign that the cognition underlying the imagined right-handed rhythmic improvisation is more internally-directed.

With respect to the beta band, we found higher activity in the right RMF and PCG for Copy compared to Improvise (see Figure 3). Higher beta power in the right dorsal lateral prefrontal cortex (which shares anatomy with the RMF) during response preparation to an anti-saccade task has also been implicated as a sign of inhibitory control (Hwang et al., 2014). In a review of functional evidence on inhibition control, Aron et al. (2014) argue that critical brain areas are right-lateralized, and furthermore propose that the dorsal lateral prefrontal cortex is involved in regulating the rules of inhibition control. Meanwhile, the right primary motor cortex, which is within the PCG, is also recognized as playing a role in action inhibition (Spierer et al., 2013), and a recent electrocortiographical study has found higher beta oscillatory power here prior to a successful stop during a no-go task (Fonken et al., 2016), thus implicating higher beta oscillatory power in this brain area as a sign of inhibition.

Inhibition can be considered a form of conscious self-monitoring. Such processing is necessary for the Copy condition of the present study as subjects must ensure their imagined rhythmic pattern accurately matches that of the presented stimulus. Conversely less self-monitoring, or disinhibition, is arguably key to the spontaneous free flowing of ideas intrinsic to improvisational cognition. Neurological evidence that improvisation involves a disinhibited cognitive state has previously been reported by Limb and Braun (2008), who similarly observed deactivation in the right dorsal lateral prefrontal cortex during free right-handed improvisation by professional jazz players. In the present study, both Multi-finger Improvise and Multi-finger Copy involve a degree of improvisational cognition pertaining to melody, as the subject is free to use any combination of notes to perform the task. However, it is only Improvise in which subjects are completely free to improvise their responses. Thus, cognition during Improvise should be more disinhibited, and may therefore explain why beta activity for Improvise was lower in frontal areas associated with inhibition control.

The present study was limited in that the time-frequency analyses used were based on average activity within relatively broad, pre-determined areas, which intrinsically lowered the spatial precision of spectral activity. However, we feel the method is justifiable and informative for this first-time, exploratory inquiry of improvisational music performance-related cognition in MEG. Meanwhile, the lack of significant results for the Single-finger task may indicate that, for the experienced musicians in this study, the cognitive burden for both Single-finger conditions was insufficient to generate observable interactions between brain activity and brain area. This may not be the case with non-musicians however, and thus further experimentation in non-musician populations is needed. Finally, the tasks in the present study were not “yorked” (Engel and Keller, 2011). In other words, subjects did not respond to stimuli produced by another subject, as was the case in the improvisational music performance study by Donnay et al. (2014). By using a “yorked” performance design, the deviation in rhythmic complexity (i.e., note count) between Improvise and Copy could have been lessened, and may have resulted in more robust differences in brain activity between the conditions.

## Conclusion

The present study is the first to successfully demonstrate the feasibility of musical improvisation experimentation in MEG. Moreover, using a novel MEG compatible MIDI keyboard, the present study was able to differentiate spectral-spatial differences due to mental imagery of improvisational music performance, with greater theta activity in left temporal rhythm production and communication areas, greater alpha activity in left sensorimotor and premotor areas, and less beta-activity in areas associated with inhibition control. These findings highlight the communicative nature of the improvisational style used, and support the notion that production of novel auditory content may be facilitated by a more internally-directed, disinhibited cognitive state. In all, the present study marks an important first step for neuromagnetic research regarding improvisational music cognition, and will hopefully serve as a foundation for future studies and analyses that compare brain activity between musicians and non-musicians, and investigate the effects of improvisational music training and therapy.

## Author Contributions

JB was the primary contributor for all aspects of the study, including experimental design and execution, data analysis and writing and revising the manuscript. YT contributed to experimental design, data analysis and editing the manuscript. SK and KY contributed to data analysis and editing the manuscript.

## Conflict of Interest Statement

The authors declare that the research was conducted in the absence of any commercial or financial relationships that could be construed as a potential conflict of interest.

## Footnotes

^1^http://neuroimage.usc.edu/brainstorm
